# Tracking Alcohol Consumption Over Time

**Published:** 2003

**Authors:** Thomas K. Greenfield, William C. Kerr

**Affiliations:** Thomas K. Greenfield, Ph.D., is a senior scientist and director, and William C. Kerr, Ph.D., is an associate scientist, both at the Alcohol Research Group, Public Health Institute, Berkeley, California

**Keywords:** research and evaluation method, research quality, aggregate AOD (alcohol and other drug) consumption, cross-sectional study, survey, longitudinal study, population study, trend, statistical modeling, epidemiology

## Abstract

Researchers are tracking long-term changes in alcohol consumption and related behaviors or outcomes in order to detect trends in the entire population or certain subgroups, test models of alcohol-related outcomes, and understand the consequences of interventions. Such analyses must consider the complexity of typical lifetime consumption patterns. Major approaches to measuring alcohol consumption over time include aggregate measures of consumption (e.g., sales data), cross-sectional surveys, and longitudinal or panel surveys. When analyzing the data, researchers must try to ensure the comparability of measurements over time. The stability of various measures and the potential for combining different types of data are also important considerations when tracking alcohol consumption over time. If these requirements are met, the regular collection of data on aspects of alcohol consumption will greatly increase researchers’ understanding of the forces influencing a population’s alcohol consumption and its consequences.

Epidemiologists not only monitor current alcohol consumption and its consequences (e.g., traffic crashes) as well as other alcohol-related behaviors, they also analyze long-term trends in these variables. This article summarizes some of the goals of such trend analyses, reviews three major types of trend measurements, and explores the comparability of such measurements over time. The article also discusses the stability of various measures and the possibilities for combining different types of data.

## Epidemiological Goals of Tracking Alcohol Consumption Over Time

Regular and detailed monitoring of a nation’s alcohol consumption has several benefits. First, each measurement provides a current portrait of drinking practices, and repeated measurements allow for early detection of trends in drinking patterns both for the entire nation and for population subgroups. Certain subgroups, such as ethnic minorities whose health problems and access to health care may differ from those of the majority of the population, may warrant particular attention in such analyses because they may be more vulnerable to alcohol-related and other health problems.

Second, by tracking alcohol consumption over time, investigators obtain the information needed to test temporal models of alcohol consumption behaviors and related outcomes, such as alcohol-related mortality and morbidity, including the development of alcohol dependence.

Third, close monitoring of the consumption patterns of a population subgroup or of people residing in a given geographic area may be vital for understanding other alcohol-related social harms, such as spousal violence, urban blight, or poor academic performance in a school setting.

Fourth, routine monitoring with rapid reporting allows investigators to detect changes in measures such as the prevalence of heavy-drinking episodes at an early stage, providing a basis for the planning and targeting of prevention programs. Finally, repeated measurements of alcohol consumption are needed to estimate the effectiveness of policy changes related to alcohol consumption, such as alcohol excise taxes and availability controls, increased accessibility of or entitlements to treatment, enactment or enforcement of drunk-driving laws, welfare reform, advertising, and health education. Particularly in light of ongoing economic and cultural changes that also affect drinking, repeated monitoring of alcohol consumption is helpful for understanding the effects of specific interventions and policy measures.

Changes over time occur at two levels: the individual level and the population level. Changes at the individual level can be monitored by diary, longitudinal, or retrospective surveys in which data are collected for the same individuals at different points in time. Changes at the population level can be collected by evaluating aggregate or survey data collected at periodic intervals for a population whose definition remains constant but whose members change over time (e.g., people age 14 and older, or adults age 18 and older) (World Health Organization [WHO] 2000).

## U.S. Alcohol Consumption Patterns

When tracking alcohol consumption patterns both for individuals and for an entire population, researchers must consider the complexity of typical lifetime consumption patterns. For each person, alcohol consumption is zero (or minimal) before the initiation of drinking and then follows a sporadic pattern during which drinks are consumed at varying rates during certain hours of a varying number of days per week or month. Many drinkers, however, go through cycles in terms of whether they drink at all, how much they drink, and what type of beverage they consume. These cycles can occur both weekly (e.g., consumption during the weekends versus weekdays) and seasonally (e.g., the winter holiday season versus the rest of the year). Changes, whether cyclic or not, may also occur over the life course.

Some people will never drink (lifetime abstainers) or will give up drinking entirely (ex-drinkers). In epidemiological surveys, ex-drinkers also are classified as “current abstainers” if they have not consumed an alcoholic beverage within a given reference period (e.g., the previous 12 months). (For more information on epidemiological surveys and the classification of respondents, see the article by Dawson in this issue.) Many Americans drink only a few times per year, whereas others drink frequently, and some drink virtually every day for several years, or more.

It is also important to assess the amount of alcohol consumed. For example, one should distinguish frequent drinkers whose consumption remains within epidemiologically based safe-drinking guidelines (Dawson 2000)—such as *The Dietary Guidelines for Americans* established by the U.S. Department of Agriculture and U.S. Department of Health and Human Services—from drinkers whose consumption intermittently or often exceeds those standard amounts and whose drinking behavior is therefore considered risky. The dietary guidelines for Americans recommend that men should consume no more than two drinks per day and women no more than one drink per day (Dufour 2001). Consumption of five or more (5+) drinks per day has consistently been associated with various acute and chronic adverse consequences (Midanik et al. 1996). Thus, monitoring the population rates of heavy drinking is a public health priority.

Longitudinal studies following participants over extended periods of time (Fillmore et al. 1979; Temple and Fillmore 1985) have established that drinking patterns vary not only among people but also, for most people, over a lifetime. The acute and chronic effects of alcohol consumption depend on numerous factors in addition to the degree or chronicity of heavy drinking. These factors include genetic or biological sensitivity to alcohol (e.g., physiological differences in body size and the ability to metabolize alcohol), the timing and amount of food intake when drinking, the intake of other drugs, and the activities a person undertakes (e.g., driving or operating machinery). The context in which alcohol consumption occurs also plays a role; for example, given the same intake, drinking at bars may be more risky than drinking at home, because people who drink in public settings frequently drive afterward.

When tracking a group’s or individual’s alcohol consumption through surveys, researchers must keep in mind that, during any given survey period, all of these personal characteristics, life circumstances, and current and past drinking patterns interact to determine the survey result. Accordingly, interviews of a sample of survey participants generate what might be considered a cross-sectional distribution of consumption at that point in time or during that period.^1^

Most research questions investigate aggregates of consumption over the survey’s reference period (e.g., a week, month, year, or longer) (for more information on widely used measurement approaches, see the article in this issue by Dawson). For population samples, aggregation of data also occurs across individuals. In theory, it is possible to avoid this aggregation and record each drink and the time over which it is consumed, using strategies such as self-monitoring with drinking diaries, observations in settings such as bars, interactive voice-response systems for collecting daily intake, or real-time monitoring of blood alcohol levels using sensors. Although such detailed monitoring may be useful for validating the measures used for aggregate analyses, for an entire population or even a representative sample, it is generally not feasible and for many purposes not even desirable.

## Major Approaches to Measuring Alcohol Consumption Over Time

### Aggregate Measures of Consumption

Epidemiologists usually are interested in consumption volume summarized across individuals, yielding a group or subgroup’s total or average amount of alcohol consumed. The average alcohol consumption can be estimated from aggregate-level data (e.g., based on sales or taxation sources) and from individual-level survey data. Although aggregate-level data may be easier to obtain, surveys allow assessment of other aspects of drinking as well, such as the prevalence of heavy drinking or of alcohol-related problems during the reference period. Although analyses of average alcohol consumption obscure a great deal of information (e.g., the proportion of heavy versus light drinking), they offer the broadest measure of a group or population’s consumption. The average consumption across a population always changes more smoothly than individual measurements, because people vary their drinking in different ways (e.g., increasing, decreasing, or fluctuating consumption). Consequently, average consumption generally can be estimated more accurately than any individual’s particular drinking pattern.

A commonly used measure of aggregate alcohol consumption is the per capita consumption of members of a larger group (e.g., a State or Nation) within a given time period (e.g., a year, a month, or a quarter). Monthly and quarterly aggregate measures will be subject to seasonal patterns—such as increased beer consumption in the summer and increased liquor consumption in the November and December holiday season—that may distort the finding. Even shorter reference periods would further increase the risk of biases attributable to weekly and seasonal cycles. Accordingly, researchers typically favor annual reports.

To determine aggregate measures of consumption, such as the annual per capita consumption by a population, researchers determine the total amount of alcohol sold in that population during the reference period and divide it by the number of potential (not actual) drinkers. In the United States, potential drinkers are typically defined as all people age 14 and older. Acknowledging that many adolescents drink, albeit illegally, this lower age cutoff is designed to include the majority of those population groups that potentially contribute to alcohol consumption (although some people begin drinking even earlier). In the United States, these aggregates are separately measured for the broad categories of beer, wine, and spirits and for the populations of each State as well as the Nation as a whole. These per capita estimates based on sales, taxation, or industry-based shipment data are a major component of the effort to track alcohol consumption over time. The per capita estimates are thought to be more accurate than self-reported survey data and have been collected continuously for many years.

Several factors influence the accuracy of per capita consumption data. Data sources for these estimates usually are records of sales or tax receipts if available, but they also include other industry documents and monthly data on shipments from wholesale warehouses (Nephew et al. 2000). These analyses omit, however, self-imported alcohol as well as homemade or illegally produced alcohol (Giesbrecht et al. 2000). In addition, alcohol is sold at a later date than that shown in shipment data, so the timing of consumption is unknown. These discrepancies between the time of shipment, sale, and consumption make yearly data inherently more accurate than monthly data.

Inaccuracies in per capita consumption data also derive from the populations included in the analyses. For example, estimates generally are based on a State or country’s census of its population, but not all of these people drink, and the per-person average therefore includes abstainers. Moreover, residents of a particular area are generally not the only drinkers of the alcohol sold in that area; tourists, other visitors, and military personnel not included in population counts will drink some of the alcohol or may take alcohol back to their State or country for later consumption. Similarly, a State’s residents will drink when traveling and may make purchases in other States for consumption at home. These variations are usually small but can be large in some States, such as Nevada and New Hampshire. Also, not all alcohol sold is actually consumed; a portion may be spilled, left untouched, spoiled, or otherwise wasted. Furthermore, shifts in the demographic composition of the population age 14 or older may affect aggregate levels of consumption. For example, an aging population may show a decrease in overall consumption even if the age-specific rates are not changing (in populations where older drinkers drink less than younger drinkers).

Per capita consumption generally is expressed in terms of grams or liters of absolute alcohol consumed during the reference period; however, the alcohol content of the beer, wine, and spirits that make up the aggregate varies across the beverage groups, over time, and even within a brand. For example, studies found that the average alcohol content of spirits as determined through Federal tax collection and sales volume data fell from about 45 percent in the early 1960s to about 38 percent in the mid-1980s (Kling 1989, 1991).

Because the alcohol content of beer and wine varies across the range of products, differences in consumer choices can result in variations in average alcohol content across States and over time. These variations can be illustrated with the example of beer consumption. Whereas a typical light beer has an alcohol content of 4.2 percent, most premium beers contain 5 percent alcohol, and malt liquor, ice beers, and many microbrewery beers contain 5 to 7 percent alcohol or more (Adams Business Research 2001). If investigators use a constant mean alcohol content for all States and all years, such as the 4.5 percent used in National Institute on Alcohol Abuse and Alcoholism Surveillance Reports (Nephew et al. 2000), differences across States and over time in the types of beer consumed will result in mismeasurement. For example, in 2000, Iowa consumers drank 65.4 percent light beer, 0.5 percent malt liquor, 2.7 percent ice beer, 3.4 percent microbrewed/specialty beer, and 28 percent popular and premium beer. New York consumers drank 32.8 percent light beer, 4.5 percent malt liquor, 6.7 percent ice beer, 7.7 percent microbrewed/specialty beer, and 48.3 percent popular and premium beer (Adams Business Research 2001).

Kerr and Greenfield (2003) have estimated the average alcohol content of beer sold in the United States in 1995 and 2000, finding that significant differences existed across States. When they applied those estimates, the researchers found that the relative ranking of beer as a source of alcohol consumption changed for 28 States (in some cases by several places) compared with other measurement approaches. These findings establish the importance of improving the accuracy of alcohol content estimates for the beverage groups in the United States.

Despite these issues, per capita beverage-specific and total alcohol consumption are the standard measures for tracking consumption over time and for cross-State and cross-national comparisons. One reason for this preference is that many important economic, social, demographic, and epidemiologic variables are available on comparable levels of aggregation. As a result, by using these variables and potentially controlling for other factors, researchers can construct time-series models both of alcohol consumption and of alcohol-related outcomes.

### Cross-Sectional Surveys

Cross-sectional surveys offer snapshots of the range of measured consumption behaviors during a particular reference period preceding the day of the interview (for more information on measurement issues for such surveys, see the article by Dawson in this issue). Using cross-sectional surveys, researchers can assess lifetime patterns of consumption, although recall over long periods may be prone to systematic biases related to age as well as to current drinking (Lemmens 1998). Cross-sectional trend studies—repeated surveys of distinct samples—are based on two or more comparably measured consumption assessments over a certain period. (Similar analyses can be conducted using longitudinal panel designs that involve repeated assessments of the same subjects, as discussed in the next section.) In national surveys, measurements for such trend studies are usually taken several years apart. For example, the Alcohol Research Group conducts the National Alcohol Survey (NAS) at intervals of approximately 5 years (Greenfield et al. 2000).

Surveys are essential for assessing variables that are not available from aggregate-level data, such as drinking quantities and frequencies (see the table). Surveys also provide vital public health information on other aspects of alcohol consumption, including the following:

- The locations where a respondent consumes alcoholic beverages (e.g., at home or in restaurants or bars)
- The types of beverages a respondent prefers
- Complex diagnostic assessments, such as the criteria for alcohol dependence specified in the American Psychiatric Association’s (1994) *Diagnostic and Statistical Manual of Mental Disorders, Fourth Edition* (DSM–IV)
- The occurrence of alcohol-related consequences, such as job or family difficulties, hospital or emergency room visits, and other adverse effects.

As with other measurement methods, however, the accuracy of reporting is compromised when the respondent is asked to look farther back in time, requiring investigators to weigh the wish to avoid imprecise recall against the desirability of measuring longer-term patterns.

Series of cross-sectional surveys are relatively easy to implement because they do not require investigators to trace participants over time or to obtain consent for participation in further followups. If the methods are highly similar from survey to survey, with equivalent sampling and measurement approaches, these series can allow researchers to track a population’s patterns of alcohol use (Greenfield et al. 2000), alcohol-related problems (Midanik and Greenfield 2000), and factors that may influence these problems (Greenfield and Room 1997). Cross-sectional surveys also can identify special populations and oversample^2^ them to ensure sufficient statistical power to analyze drinking problems and shifts in drinking patterns in those populations over time. Special populations that may be monitored with trend studies include age groups, such as youth or the elderly; ethnic groups, such as African Americans and Hispanics (Caetano and Clark 1998); groups based on drinking behaviors, such as alcoholics, heavy drinkers, or bar patrons; and other groups, such as homeless people, military personnel, or women (Wilsnack 1996). Trends among these demographic subgroups cannot be derived from aggregate trend data. Furthermore, survey data allow analysts to account for shifts in the age, ethnicity, gender, or other demographic characteristics of the population when considering the sources of change in alcohol consumption measures.

The primary problem associated with cross-time comparisons is the issue of consistent measurement, which is affected by the subjective nature of self-reporting alcohol consumption and alcohol-related problems. Methodological studies are beginning to document the performance of standard measures used in cross-sectional as well as longitudinal studies and in different interview modes, such as face-to-face, mail, and telephone interviews (Greenfield 2000). A secondary problem—that is, declining response rates over time—leads to questions as to whether the same population is actually being measured at each time.

### Longitudinal Surveys (Panel Surveys)

Longitudinal or panel surveys allow researchers to study the development of individual consumption patterns (including those of members of ethnic groups) over extended periods and to identify subgroups of drinkers, such as chronic heavy drinkers (Caetano and Kaskutas 1995). Longitudinal designs are also valuable for examining trends in consumption patterns and associations between these patterns and related problems over time (Caetano and Kaskutas 1996; Muthen and Muthen 2000). Although such relationships are also studied in periodic cross-sectional surveys, longitudinal designs help establish relationships at the individual level and allow researchers to make stronger causal attributions. Finally, these studies allow for tracking of mortality and morbidity outcomes, particularly outcomes related to chronic consumption (Shaper and Wannamethee 1998).

Longitudinal surveys are similar to cross-sectional surveys in the types of behaviors and outcomes they measure. Compared with cross-sectional analyses, however, longitudinal studies add measurement opportunities across time from as little as a month to many years apart. Prospective surveys offer particularly accurate insight into consumption over time because they avoid the recall problems regarding past consumption that are associated with retrospective lifetime measures. Finally, longitudinal surveys can uncover longer-term changes in patterns of consumption and relate these changes to individual and societal factors.

One potential problem with longitudinal studies is that often they are not representative of the general population on all measurement points because they may suffer from considerable attrition or from the researchers’ inability to locate and secure reinterviews with still-living participants.^3^ If longitudinal surveys are representative of the general population at the first measurement, however, and if researchers can take into account the effects of attrition, these designs offer a picture of consumption trends that augments and complements representative cross-sectional surveys. The issues related to measuring aggregations of consumption are important here as well. Foremost, measures and methods must be comparable with each other, but even then the choice of measure itself can also influence whether relevant long-term patterns will be detected. For example, different measures have different sensitivities (or accuracy in assessing actual consumption) and thus have been found to account for differing amounts of the total consumption as measured by sales figures (i.e., they have different coverage rates^4^).

## Comparability of Measurements Over Time

Aggregate data are generally thought to be the most comparable over time. Despite the concerns mentioned above regarding the accuracy of aggregate data, usually no large-scale systematic changes occur in the way aggregate consumption is measured. Data from cross-sectional or longitudinal surveys, in contrast, can be more variable, because many opportunities exist for mismeasurement and changes over time. For example, the wording and order of the questions, the survey mode (e.g., face-to-face or telephone interview, self-completed booklet, Web-based survey, or mailed form), sampling methods (e.g., multistage household probability versus random-digit dial samples), and the general attitude of the population toward surveys and the resulting response rates can change over time. Even the concepts of a “standard drink,” alcohol problems, or drunkenness can change over time through cultural redefinition. In some cases, such redefinition results from policy changes such as Prohibition, the increase of the minimum drinking age to 21, or the lowering of the legal blood alcohol concentrations for driving to 0.08 percent. All of these variations affect the accuracy and comparability of surveys by introducing both systematic and random errors. Studies of methodological effects and of ways to adjust for these effects are one promising partial remedy (Greenfield 2000).

To avoid or minimize these problems, survey series rely on large, representative samples and attempt to keep methods equivalent or include methodological checks. However, many surveys used in the analysis of alcohol-related behaviors and outcomes are conducted primarily for other purposes and contain only one or two questions that attempt to measure alcohol consumption. Even in these surveys that do not focus on alcohol consumption, consistency in the questions, interview structure, and sampling is desirable for comparisons over time. This need for comparability with past surveys, however, conflicts with the desire to improve measurement by optimizing survey methods, particularly for repeated cross-sectional surveys such as the NAS. To address this conflict, researchers have found ways to ask questions in their original formats yet improve precision by adding certain questions in later surveys. For example, a question without a reference period, such as “How often do you usually have any kind of beverage containing alcohol—whether it is wine, beer, whiskey, or any other drink?” may be asked in two subsequent surveys. In the later, “improved” questionnaire, the item may be followed up by the question, “Think back over the last year, since [current date last year]; did you have a whole drink of any alcoholic beverage like wine, beer, or liquor in these last 12 months?” The first item may be used alone to track frequency of consumption, whereas the two-question format of the later survey allows for improved precision and provides a sensitivity check on the effects of omitting the reference period.

## Stability of Various Measures and Possibilities for Combining Different Types of Data

All approaches to tracking alcohol consumption (i.e., aggregate analyses, repeated cross-sectional surveys, and longitudinal studies) will detect changes over time, but the types and extent of changes detected depends on the type of study. Among the various levels and types of aggregate data, yearly aggregates tend to change slowly and may obscure a great deal of instability in the consumption levels of individual drinkers. Similarly, repeated cross-sectional surveys may find small changes in the overall distribution of the volume of consumption and the number of heavy drinkers or abstainers, but this relative stability may mask individual change. Conversely, longitudinal studies may identify individuals who increase or decrease their consumption considerably within a population that exhibits a stable level of consumption overall (Kerr et al. 2002).

To identify population trends in alcohol consumption that imply a collective shift in drinking patterns, researchers must look at aggregate data, because individual changes in drinking behavior may balance one another out. The types of trends that can be detected, however, depend on the level of aggregation. Daily consumption trends show weekend spikes, and weekly or monthly trends may display seasonal patterns; only annual trends average all of these changes to reveal substantive secular trends in the amount of drinking. For example, long-term analyses of annual aggregate data demonstrated that with the exception of a brief period during World War II, per capita alcohol consumption in the United States rose relatively consistently from the repeal of Prohibition in the 1930s until approximately 1980 (see the figure). Subsequently, per capita consumption declined until approximately 1995, after which it began to increase again slightly (Greenfield et al. 2000; Nephew et al. 2000).

The difficulty in tracking consumption over time is to balance highly changeable individual drinking patterns with a population’s characteristic consumption, such as average daily volume over the last year. The relationships between the different types of measures and forms of data highlight the importance of tracking as many measures as possible in order to achieve a clearer picture of the changes occurring in the population. In the past, only aggregate sales data have been available on a continuous basis. In recent years, however, several national surveys tracking alcohol consumption and related problem trends over time have become available (see the sidebar).

**Surveys Tracking Alcohol Consumption**

In recent years, several surveys that track alcohol consumption and a variety of other alcohol-related measures have become available. These surveys include the following:

- *The Gallup Survey*. Since 1939, this survey has frequently, but sporadically, measured the proportion of the population who drink alcohol. In 1950 and 1964, and more frequently since 1974, the survey has assessed whether drinking caused family problems. More information is available at www.gallup.com.
- *The National Alcohol Survey (NAS)*. This survey, conducted by the Alcohol Research Group, has measured many aspects of alcohol consumption, associated problems, and use of treatment approximately every 5 years since 1959. The latest surveys were conducted in 1979, 1984 (with longitudinal followup in 1992), 1990, 1995, and 2000. The 1984, 1995, and 2000 surveys oversampled African American and Hispanic respondents. More information is available at www.arg.org.
- *The Behavioral Risk Factor Surveillance System* (BRFSS). Since 1984, this system has collected State-level representative measures of past-month abstinence, frequency of drinking, usual quantity per occasion, frequency of five or more drinks on one occasion, and frequency of drunk driving. The BRFSS covered 15 States when it began in 1984, increasing to 40 States in 1989, 48 States in 1991, 50 States in 1993, and adding the District of Columbia in 1996. Thirteen States have participated in all years. Alcohol consumption questions were included in the core survey every year from 1984 to 1993 and in 1995, 1997, and 1999. An optional alcohol consumption module was used by 17 States in 1996, 12 States in 1998, and 10 States in 2000. The objective of the BRFSS is to collect uniform, State-specific, and State-representative population data on risk behaviors and preventive health practices. More information is available at www.cdc.gov/nccdphp/brfss/.
- *The National Health Interview Survey (NHIS)*. Since 1997, this annual survey has included questions on past-year and lifetime abstinence, past-year usual quantity, usual frequency, and frequency of five or more drinks. Before 1997, alcohol questions were included only sporadically. More information is available at www.cdc.gov/nchs/nhis.htm.
- *The Monitoring the Future (MTF) Survey*. Since 1975, this annual survey has tracked the national prevalence among 12th grade students of monthly drinking and having five or more drinks on one occasion in the 2 weeks preceding the survey. Since 1991, it has also included 8th and 10th grade students and has added a question on the monthly prevalence of having been drunk. More information is available at www.monitoringthefuture.org.
- *The National Household Survey on Drug Abuse* (*NHSDA)*. This annual survey has collected information on alcohol use since 1974, and since 1994 it has included questions on lifetime, yearly, and 30-day abstinence; yearly and 30-day drinking frequency; 30-day usual quantity; 30-day frequency of five or more drinks; and yearly frequency of “getting very high or drunk” from alcohol. More information is available at www.samhsa.gov/oas/nhsda.htm.

—Thomas K. Greenfield and William C. Kerr

## Conclusions

In their efforts to track alcohol consumption and related variables in a population over extended periods of time, researchers rely on aggregate data, cross-sectional surveys, and longitudinal or panel studies. Aggregate alcohol consumption data are used primarily to establish models of alcohol-related outcomes, such as mortality and morbidity from alcohol-related causes, injury rates, or traffic fatalities. These data also allow for the development of models linking aggregate consumption with demographic and policy variables, such as tax rates, minimum drinking age limits, and warning labels. These models are based either on time series of the relevant variables from a single population (e.g., the entire United States) or on cross-sectional data from several populations (e.g., different States). Identifying time-series relationships is complicated by a number of issues, including the possibility of spuriously correlated trends (see Norstrom and Skog 2001).

The regular collection of survey measures on aspects of alcohol consumption will increase researchers’ understanding of the dynamic forces involved in a population’s changing consumption pattern. By tracking alcohol consumption through surveys, researchers can begin to answer questions such as the following:

- Do changes occur primarily among current drinkers or among people moving in or out of drinking?
- Are the heaviest drinkers relatively immune to economic or cultural forces that affect other drinkers (i.e., do they appear to form a separate group)?
- Do the differences in consumption widely observed between men and women converge over time?

Sometimes it is difficult to determine the sources of changes in individual consumption patterns observable in repeated surveys. Such changes may be attributable to a person’s age (i.e., a maturation effect) to the period (i.e., societal conditions prevailing at the time of the survey), or to the birth cohort (e.g., the cultural ambience related to drinking during the formative years, when many people begin to drink). Age-period-cohort (APC) models are one strategy that analysts can use with series of surveys in an effort to distinguish these various possibilities. Despite these caveats, however, efforts to track alcohol consumption with repeated representative surveys that employ consistent measures and procedures can help answer important epidemiological questions.

**Figure:**
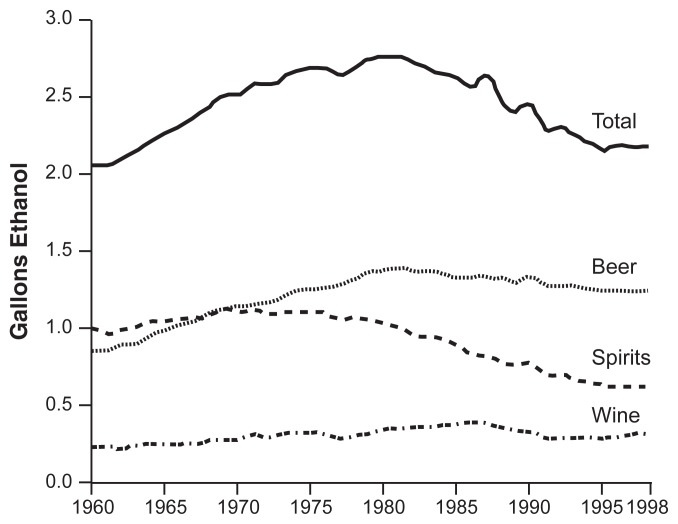
Per capita consumption of beer, wine, and spirits, and total alcohol consumption in the United States, 1960–1998. SOURCE: Adapted from Nephew et al. 2000.

Prospective longitudinal surveys offer the best opportunity to link individual characteristics to the time course of alcohol use and alcohol-related problems as well as to reveal the causal sequences involved. Although cross-sectional surveys can be useful for this purpose, longitudinal designs best address questions regarding the individual’s response to policies (e.g., taxation changes) or interventions (e.g., improved availability of treatment services). Longitudinal studies also are important for estimating alcohol-related morbidity and mortality as well as other alcohol-related problem outcomes over longer periods. These studies offer multiple live measures, which have been found to be more accurate than retrospective assessments and which can be linked with outcomes assessed at followup.

The three types of data used in tracking alcohol consumption over time complement one another for many purposes. Data from representative surveys can be aggregated for attributing total alcohol consumption to different types of drinkers or for assessing the impact of individual-level changes on aggregate consumption. Similarly, aggregate-level statistics (e.g., State-level) can be attached to individual observations in surveys to represent societal effects on individual behavior. Hierarchical modeling is essential for such multilevel approaches. These modeling approaches distinguish variability owing to individual influences from those owing to group-level influences. For example, alcohol use among adolescents may differ among the students within a classroom, but certain influences on the adolescents’ likelihood of drinking may occur at the classroom or school level. In models attempting to disentangle the effects of age, period, and cohort (i.e., APC decompositions), researchers can combine repeated cross-sectional surveys and longitudinal surveys to allow for more accurate identification of time-related effects. For example, although longitudinal data already permit researchers to assess drinking behaviors of individuals in each birth cohort and to follow them at multiple ages and in different periods, multiple self-report measurements on the same individuals may result in sensitization or reactivity. This means that the results of earlier assessments influence the results of later assessments. Cross-sectional surveys do not introduce this problem. In general, each method of tracking alcohol consumption and related problems over time has some trade-offs; consequently, the use of multiple methods in such studies is often desirable.

## Figures and Tables

**Table 1:** Drinking Trends From Repeated Cross-Sectional Surveys—Examples of Measures not Available From Aggregate-Level Data

	1984 (*n* = 5,221)	1990 (*n* = 2,058)	1995 (*n* = 2,178)	χ^2^† 1984 vs. 1990	χ^2^ 1990 vs. 1995
**All respondents, % (SE)**					
Current drinking	69.4 (1.6)	65.0 (1.4)	64.6 (1.6)	4.04*	0.03
Wine	51.2 (1.8)	43.6 (1.5)	42.7 (1.9)	10.65**	0.20
Beer	51.5 (1.3)	45.2 (1.4)	48.0 (1.6)	9.61**	2.19
Spirits	51.8 (1.8)	43.5 (1.3)	42.6 (1.7)	13.85***	0.07
Weekly drinking	35.9 (1.5)	29.0 (1.2)	29.2 (1.3)	13.90***	0.12
5+ drinks ever in prior year	30.0 (1.2)	28.6 (1.2)	27.6 (1.4)	0.66	0.42
5+ drinks weekly in prior year	6.1 (0.6)	3.9 (0.5)	4.5 (0.6)	8.66**	0.93
**Current drinkers, mean (SE)**				**t**†† **1984 vs. 1990**	**t** **1990 vs. 1995**
Total drinking days	109.7 (4.6)	82.9 (3.9)	87.7 (3.9)	4.00***	0.05
Wine	39.8 (2.5)	39.3 (3.0)	39.5 (3.0)	0.13	0.05
Beer	95.8 (4.1)	72.2 (3.9)	75.4 (3.6)	4.19***	0.59
Spirits	34.1 (1.9)	31.5 (1.9)	26.2 (1.9)	0.98	1.98*
Total heavy drinking days	19.3 (1.5)	13.2 (1.2)	13.2 (1.3)	2.71**	0.07
Wine	1.9 (0.4)	1.5 (0.4)	1.0 (0.2)	0.63	0.99
Beer	13.9 (1.1)	9.4 (0.9)	10.5 (1.0)	2.74**	0.91
Spirits	3.7 (0.5)	2.6 (0.5)	1.9 (0.3)	1.37	1.26

* *p* < 0.05;

** *p* < 0.01;

*** *p* < 0.001

† The chi-square statistic is used to test a hypotheses concerning the probability of whether a behavior or characteristic found in a sample—or in this case, the change in that behavior or characteristic found from one sample to another—is found to the same degree in the population as a whole.

†† The *t* test assesses whether the means of two groups are statistically different from each other.

NOTE: This table is based on weighted data obtained from U.S. respondents participating in the 1984, 1990, and 1995 National Alcohol Surveys. The table displays percentages and means, as well as the standard error (SE).

SOURCE: Adapted from Greenfield et al. (2000).

## Footnotes

1 In practice, surveys are conducted over extended periods of time, but the principle nevertheless holds.

2 Oversampling means including more members of a special population in the survey than would correspond to that population’s proportion in the total population.

3 Nonresponse is an increasing problem for both cross-sectional and longitudinal surveys.

4 The coverage rate is the ratio of survey-measured to aggregate-measured consumption.
